# Antimicrobial Blue Light Inactivation of Polymicrobial Biofilms

**DOI:** 10.3389/fmicb.2019.00721

**Published:** 2019-04-09

**Authors:** Raquel Ferrer-Espada, Xiaojing Liu, Xueping Sharon Goh, Tianhong Dai

**Affiliations:** ^1^Wellman Center for Photomedicine, Massachusetts General Hospital, Harvard Medical School, Boston, MA, United States; ^2^Vaccine & Immunotherapy Center, Massachusetts General Hospital, Harvard Medical School, Boston, MA, United States

**Keywords:** antimicrobial blue light, polymicrobial, biofilm, *Pseudomonas aeruginosa*, *Staphylococcus aureus*, *Candida albicans*, CDC biofilm reactor, endogenous photosensitizer

## Abstract

Polymicrobial biofilms, in which mixed microbial species are present, play a significant role in persistent infections. Furthermore, polymicrobial biofilms promote antibiotic resistance by allowing interspecies transfer of antibiotic resistance genes. In the present study, we investigated the effectiveness of antimicrobial blue light (aBL; 405 nm), an innovative non-antibiotic approach, for the inactivation of polymicrobial biofilms. Dual-species biofilms with *Pseudomonas aeruginosa* and methicillin-resistant *Staphylococcus aureus* (MRSA) as well as with *P. aeruginosa* and *Candida albicans* were reproducibly grown in 96-well microtiter plates or in the CDC biofilm reactor for 24 or 48 h. The effectiveness of aBL inactivation of polymicrobial biofilms was determined through colony forming assay and compared with that of monomicrobial biofilms of each species. aBL-induced morphological changes of biofilms were analyzed with confocal laser scanning microscopy (CLSM) and scanning electron microscopy (SEM). For 24-h old monomicrobial biofilms formed in 96-well microtiter plates, 6.30-log_10_ CFU inactivation of *P. aeruginosa*, 2.33-log_10_ CFU inactivation of *C. albicans* and 3.48-log_10_ CFU inactivation of MRSA were observed after an aBL exposure of 500 J/cm^2^. Under the same aBL exposure, 6.34-log_10_ CFU inactivation of *P. aeruginosa* and 3.11-log_10_ CFU inactivation of *C. albicans* were observed, respectively, in dual-species biofilms. In addition, 2.37- and 3.40-log_10_ CFU inactivation were obtained in MRSA and *P. aeruginosa*, dual-species biofilms. The same aBL treatment of the biofilms developed in the CDC-biofilm reactor for 48 h significantly decreased the viability of *P. aeruginosa* monomicrobial and polymicrobial biofilm when cocultured with MRSA (3.70- and 3.56-log_10_ CFU inactivation, respectively). 2.58-log_10_ CFU inactivation and 0.86-log_10_ CFU inactivation was detected in MRSA monomicrobial and polymicrobial biofilm when cocultured with *P. aeruginosa*. These findings were further supported by the CLSM and SEM experiments. Phototoxicity studies revealed a no statistically significant loss of viability in human keratinocytes after an exposure to 216 J/cm^2^ and a statistically significant loss of viability after 500 J/cm^2^. aBL is potentially an alternative treatment against polymicrobial biofilm-related infections. Future studies will aim to improve the efficacy of aBL and to investigate aBL treatment of polymicrobial biofilm-related infections *in vivo*.

## Introduction

Microorganisms are prone to forming biofilms, surface-associated microbial communities that are extremely resistant to antimicrobials and the immune system (Costerton et al., 1999). Moreover, biofilm-forming microorganisms are far more resistant to antimicrobials than organisms in suspension (Dunne, 2002). It is reported that biofilms are involved in up to 65% of infections where they often lead to severe illness with a prolonged hospital stay, increased costs, and high mortality (La Fuente-Núñez et al., 2014).

Most diseases have previously been characterized as monomicrobial in nature, likely due to the extensive use of culture-dependent isolation techniques. Now, with the advent of culture-independent analysis methodologies, several are becoming increasingly recognized as polymicrobial infections (Peters et al., 2012). Polymicrobial biofilms, in which mixed microbial species are present, are the dominant form of microbial life in nature (Harriott and Noverr, 2011; DeLeon et al., 2014). By including mixed microbial species in a single community, biofilms obtain numerous advantages, such as an enlarged gene pool with more efficient DNA sharing, quorum sensing systems, passive resistance, metabolic cooperation, and many other synergies (Wolcott et al., 2013). For instance, the gram-negative pathogen *Pseudomonas aeruginosa* is commonly found in mixed infections with the polymorphic fungus *Candida albicans* or the gram-positive bacterium *Staphylococcus aureus* (Harriott and Noverr, 2011; DeLeon et al., 2014). As such, *P. aeruginosa* and *C. albicans* are often found together in burn wound infections, contaminated catheters and chronic lung infections (Peleg et al., 2010). *P. aeruginosa* and *S. aureus*, on the other hand, have frequently been isolated from chronically infected wounds, chronic suppurative otitis media, indwelling medical devices, abnormal airways such as those in cystic fibrosis and other chronic obstructive lung diseases (Yadav et al., 2017).

*P. aeruginosa* can attach to the surface of *C. albicans* hyphae (but not yeast cells) and form dual-species biofilms (Peleg et al., 2010). Interspecies competition between *P. aeruginosa* and *C. albicans* enhances the production of virulence factors and increases mutability, and thus can alter the course of host-pathogen interactions in polymicrobial infections (Peleg et al., 2010; Trejo-Hernández et al., 2014). As a result, dual-species biofilms with *P. aeruginosa* and *C. albicans* play extensive roles in nosocomial infections and infection in immunocompromised individuals (Fourie et al., 2016).

*S. aureus* and *P. aeruginosa* are two versatile bacterial pathogens that are frequently found together in chronic wound infections (Stacy et al., 2016). Dual *S. aureus* and *P. aeruginosa* infections are more virulent and/or result in worse outcomes than the single infections caused by either species. The mutualistic and parasitic interactions drive the synergistic impact of the two species on the progression of infections (Nguyen and Oglesby-Sherrouse, 2016).

Effective therapy for tackling the antimicrobial resistance in polymicrobial biofilms is lacking. There is a pressing need for the development of new strategies against polymicrobial biofilm infections. Antimicrobial blue light (aBL) in the spectrum of 400 to 470 nm, as an innovative light-based non-antibiotic strategy, has attracted increasing attention due to its intrinsic antimicrobial effect without the involvement of exogenous photosensitizers (Dai et al., 2012; Wang et al., 2017; Gwynne and Gallagher, 2018). The proposed mechanism of action of aBL involves the natural accumulation in microbial cells of photoactivable metal-free porphyrins such as uroporphyrin, coproporphyrin, and to a lesser extent protoporphyrin (Dai and Hamblin, 2017). These endogenous porphyrins absorb the Soret band of light (405–420 nm) and are subsequently excited to the triplet state, where singlet oxygen is generated (Dai et al., 2012; Dai and Hamblin, 2017; Wang et al., 2017; Gwynne and Gallagher, 2018). Singlet oxygen rapidly reacts with a wide range of cellular macromolecules and damages proteins, lipids, DNA, and RNA (Glaeser et al., 2011; Bumah et al., 2017). Additional mechanisms of action such as prophage activation have also been proposed (Yang et al., 2017). Similar to antimicrobial photodynamic therapy (Dai et al., 2009), aBL inactivation of microorganisms is thought to be a multi-target damaging process (Maisch, 2015). As a consequence, the likelihood for the development of aBL-resistance by microorganisms is less than that of antibiotic resistance.

In the present study, we investigated the effectiveness of aBL inactivation of polymicrobial biofilms *in vitro*. Monomicrobial and polymicrobial biofilms of *P. aeruginosa*, *S. aureus* and *C. albicans* were reproducibly grown in 96-well microtiter plates or the CDC biofilm reactor. The effectiveness of aBL inactivation of polymicrobial biofilms was determined through colony forming assay and compared with that of the monomicrobial biofilms of each species. Furthermore, the morphological changes of biofilms induced by aBL were analyzed with confocal scanning microscopy (CLSM) and scanning electron microscopy (SEM).

## Materials and Methods

### Blue Light Source

For aBL irradiation, we used a light-emitting diode (LED) with peak emission at 405 nm and a full width at half maximum (FWHM) of 20 nm (M405L3, Thorlabs, United States). The irradiance on the surface of the target was measured using a PM100D power meter (Thorlabs). The distance between the LED aperture and the target was set at 3 cm for 92.6 mW/cm^2^ and 4 cm for 60 mW/cm^2^. The radiant exposure was calculated with the following equation (Radiant exposure (J/cm^2^) = Irradiance (W/cm^2^) × Exposure time (s)).

### Microbial Strains and Culture Conditions

The strains used in this study were *P. aeruginosa* IQ0046 (a clinical multidrug-resistant isolate) (Lu et al., 2018), *C. albicans* CEC 749 (Enjalbert et al., 2009), and MRSA USA300 (Vidlak and Kielian, 2016). These strains were routinely grown at 37°C and 150 rpm in Brain Heart Infusion (BHI; BioMerieux, France) broth or BHI supplemented with 15 g/L agar.

### Biofilms in 96-Well Microtiter Plates

#### Monomicrobial Biofilms

*C. albicans*, MRSA and *P. aeruginosa* were grown overnight in BHI broth. Then, the cell densities of the suspensions were adjusted to approximately 10^6^ CFU/mL for MRSA and *P. aeruginosa* and 10^5^ CFU/mL for *C. albicans*, and 100 μL aliquots were inoculated in 12 wells of 96-well microtiter plates (Wang et al., 2016). After 24 or 48 h of incubation (with renewal of the media every 24 h), the biofilms were carefully washed two times with phosphate-buffered saline (PBS; Thermo Fisher Scientific, United States). Then, three wells of untreated biofilms were scraped and pooled together with a sterile pipette tip and this procedure was repeated twice with fresh PBS and in two technical replicates. The biofilms in the other 6 wells were irradiated with aBL at exposures of 216 J/cm^2^ (60 mW/cm^2^, 60 min) or 500 J/cm^2^ (92.6 mW/cm^2^, 90 min) and the scraping procedure was repeated after aBL exposure. The total volume obtained, 600 μL, was sonicated for 5 min and the samples were diluted and processed for colony counting. The experiments were performed in triplicates for each condition.

#### Dual-Species Biofilms

The dual-species biofilms with *C. albicans* and *P. aeruginosa* were formed by mixing 10^5^ CFU/mL of *C. albicans* and 10^6^ CFU/mL of *P. aeruginosa* in 12 wells of a 96-well microplate; and the dual-species biofilms with MRSA and *P. aeruginosa* were obtained by mixing 10^6^ CFU/mL of each species. After 24 or 48 h of incubation (with renewal of the media every 24 h), the biofilms were exposed to 216 J/cm^2^ (60 mW/cm^2^, 60 min) or 500 J/cm^2^ (92.6 mW/cm^2^, 90 min) aBL, collected and sonicated. To enumerate the CFUs, sonicated dual-species biofilms with *C. albicans* and *P. aeruginosa* were plated on BHI agar supplemented with chloramphenicol (Sigma-Aldrich, United States) for the selective growth of *C. albicans* or with amphotericin B for the selective growth of *P. aeruginosa* (Clinical and Laboratory Standards Institute et al., 2002, 2011). The sonicated dual-species biofilms with *P. aeruginosa* and MRSA were plated on BHI agar supplemented with colistin for selective growth of MRSA or vancomycin for selective growth of *P. aeruginosa*. Growth recovery on selective growth media was compared with growth recovery on a general medium (BHI without antibiotics). Three independent experiments were performed for each condition. To ensure thermal effect was not involved in killing microorganisms, the temperatures of the samples were monitored using a Traceable^®^ Type K thermocouple (Thermo Fisher Scientific).

### Biofilms in the CDC Reactor

Biofilms of *P. aeruginosa*, MRSA and *C. albicans* were also developed under dynamic shear conditions using the CDC biofilm reactor (model CBR 90–1 DH, BioSurface Technologies Corporation, United States) as described elsewhere (Sánchez-Gómez et al., 2015; Fernández-Rivero et al., 2017). Briefly, 400 mL BHI (37 g/L) were inoculated at 37°C with 1 mL of an overnight culture of the microorganisms and incubated in the biofilm reactor with 150 rpm stirring. The dual-species biofilms with *C. albicans* and *P. aeruginosa* or with MRSA and *P. aeruginosa* were formed by inoculating the CDC biofilm reactor with 1 mL of each one of the strains. After 24 h, a constant flow of 10 mL/min BHI (3.7 g/L) was stablished and dense biofilms (10^7^–10^8^ CFU/cm^2^) were developed on the surface of small disks called coupons. After 24 h of further incubation under continuous flow (i.e., 48 h of incubation in total), coupons were removed from the chamber and planktonic cells were eliminated by rinsing with 1 mL sterile PBS. Then, coupons were immersed in 4 mL of sterile PBS in 3.5-cm petri dishes and irradiated using aBL with exposures of 216 (60 mW/cm^2^, 60 min) or 500 J/cm^2^ (92.6 mW/cm^2^, 90 min) and processed for colony counting.

For enumerating CFU, the biofilms were detached by scraping the coupon surface with a sterile wooden stick. Then, biofilm cells were suspended in 3 mL PBS and homogenized by 5 min of sonication (Bransonic 2510R-MT, United States) and vortexing (Thermo Fisher Scientific, United States). The aliquots were serially diluted, plated for CFU counting, and the cell densities in log_10_ CFU/cm^2^ of the biofilms attached to the coupons were calculated. In turn, the calculated cell densities in log_10_ CFU/cm^2^ allowed the determination of log_10_ CFU inactivation, which was defined as the logarithm of the ratio of the CFU/cm^2^ post-aBL exposures to the CFU/cm^2^ of the untreated control. For the enumeration of CFU in the dual-species biofilm populations, sonicated samples were plated on BHI agar supplemented with chloramphenicol or amphotericin B for selective growth of *C. albicans* or *P. aeruginosa*; and on BHI agar supplemented with colistin or vancomycin for selective growth of MRSA or *P. aeruginosa* as previously described. Growth recovery on selective growth media was compared with growth recovery on a general medium (BHI without antibiotics). Experiments were independently repeated three times in duplicate coupons and the temperatures of the samples were also monitored as previously described.

### Visualization of Biofilms Using Confocal Laser Scanning Microscopy (CLSM)

Biofilm structure and microbial viability in biofilms were also visualized using CLSM. In brief, aBL-treated (500 J/cm^2^) and untreated monomicrobial and polymicrobial biofilms grown on the polycarbonate coupons of the CDC biofilm reactor were stained with SYTO 9 and propidium iodide (PI) (Invitrogen, United States). SYTO 9 is a dye that can diffuse through the membranes of microorganisms and stain DNA. On the other hand, PI can only penetrate the cells and dye DNA when the integrity of the membrane has been compromised. As a result, SYTO9 and PI work as an indicator of viability and death of microorganisms, respectively (Stiefel et al., 2015). However, PI can only be used for evaluating death of microorganisms when the membrane is damaged. In case bacteria are killed without membrane damage, death of bacteria may be understimated using this approach. After staining, the biofilms were examined with an Olympus Fluoview FV10i CLSM using the Alexa Fluor 488 nm and the 568 nm wavelength filters and the 10 × or 60 × objectives.

### Visualization of Biofilms Using Scanning Electron Microscopy (SEM)

Scanning electron microscopy was performed to investigate the morphological changes of biofilms induced by aBL treatment. Monomicrobial and polymicrobial biofilms were grown for 48 h in the CDC biofilm reactor and then exposed to 500 J/cm^2^ of aBL (92.6 mW/cm^2^, 90 min) or left untreated. After aBL exposure, the treated and untreated coupons were washed in PBS and fixed at 4°C for 24 h in 0.1 M sodium cacodylate buffer containing 2.5% glutaraldehyde, 0.15% alcian blue, and 0.15% safranin O. The fixed biofilms were washed three times for 10 min with 0.1 sodium cacodylate buffer, infiltrated with 1% osmium tetroxide for 1 h, washed another three times for 10 min with 0.1 sodium cacodylate buffer and dehydrated from 30, 50, 70, 85, 95, to 100% ethanol, spending 10 min in each solution and three consecutive exposures in the last concentration. Then, the samples were dried using a critical-point dryer, mounted on specimen stubs, sputter-coated with 8 nm platinum and examined using a high-resolution field emission SEM Hitachi S-4800. Micrographs were acquired under high vacuum using an accelerating voltage of 2.0 or 3.0 kV.

### Examination of aBL Phototoxicity to Human Keratinocytes

To test the phototoxicity of aBL to normal human epithelial cells, a monolayer of HaCaT keratinocytes were exposed to aBL at varying exposures of 216 J/cm^2^ (60 min, 60 mW/cm^2^) and 500 J/cm^2^ (90 min, 92.6 mW/cm^2^) in a 35 mm petri dish. The viability of the cells was measured using Cell Counting Kit-8 (CCK-8) (Sigma-Aldrich) after aBL exposure. CCK-8 uses WST-8 2-(2-methoxy-4-nitrophenyl)-3-(4-nitrophenyl)-5-(2,4-disulfophenyl)-2H-tetrazolium, monosodium salt, which produces a water-soluble formazan dye upon bioreduction by cellular dehydrogenases. The absorbance was determined by the optical density at 450 nm (Molecular devices Emax).

### Statistical Analysis

Data were presented as the mean ± standard error. The differences between conditions were analyzed with a one-way ANOVA followed by Tukey’s multiple comparison test (ns: not significant, ^∗^*P* < 0.05, ^∗∗^*P* < 0.01, ^∗∗∗^*P* < 0.001).

## Results

### aBL Inactivation of Monomicrobial and Polymicrobial Biofilms Grown in 96-Well Microtiter Plates

#### 24-Hour Old Biofilms

After 24 h of incubation, the obtained monomicrobial biofilms consisted of 7.76 log_10_ CFU/mL of *C. albicans*, 8.62 log_10_ CFU/mL of MRSA and 8.87 log_10_ CFU/mL of *P. aeruginosa*.

When *C. albicans* and *P. aeruginosa* were incubated together in dual-species biofilms for 24 h, the cell densities decreased to 6.97 log_10_ CFU/mL for *C. albicans* (ns, *P* = 0.159) but increased to 9.42 log_10_ CFU/mL for *P. aeruginosa* (ns, *P* = 0.534). In the 24-hour old dual-species biofilms with MRSA and *P. aeruginosa*, the cell densities were 8.59- and 8.16-log_10_ CFU/mL for MRSA and *P. aeruginosa*, respectively (ns, *P* > 0.99 and ns, *P* = 0.263) (Table 1), in comparison to the cell densities in the respective monomicrobial biofilms.

**Table 1:** Effectiveness of antimicrobial blue light (405 nm) inactivation of biofilms grown in 96-well microtiter plates after 24 h of incubation.

Biofilm type	Strain		Irradiance (mW/cm^2^)	Irradiation Time (min)	Radiant exposure (J/cm^2^)	Biofilm cell density Log_10_ (CFU/mL)	SE	Survival bacterial counts Log_10_(CFU/mL)		SE
Monomicrobial	*C. albicans*	CEC 749	60.0	60	216	7.58	0.20	-0.35	ns	0.14
			92.6	90	500	7.94	0.07	-2.33	^∗∗^	0.18
	MRSA	USA300	60.0	60	216	8.70	0.11	-1.20	ns	0.20
			92.6	90	500	8.54	0.27	-3.48	^∗∗∗^	0.39
	*P. aeruginosa*	IQ0046	60.0	60	216	8.77	0.33	-6.55	^∗∗∗^	0.45
			92.6	90	500	8.97	0.86	-6.30	^∗∗∗^	0.36
Polymicrobial	*C. albicans*	CEC 749	60.0	60	216	6.99	0.08	-2.46	^∗∗^	0.84
			92.6	90	500	6.96	0.09	-3.11	^∗∗∗^	0.43
	*P. aeruginosa*	IQ0046	60.0	60	216	9.98	0.17	-5.67	^∗∗∗^	0.38
			92.6	90	500	8.86	0.14	-6.34	^∗∗∗^	0.20
Polymicrobial	MRSA	USA 300	60.0	60	216	8.23	0.09	-1.42	ns	0.24
			92.6	90	500	8.96	0.14	-2.37	^∗∗^	0.23
	*P. aeruginosa*	IQ0046	60.0	60	216	8.05	0.08	-3.94	^∗∗∗^	0.09
			92.6	90	500	8.28	0.03	-3.40	^∗∗∗^	0.34

*ns, not significant; ^∗∗^P < 0.01, ^∗∗∗^P < 0.001. SE, standard error. The differences between untreated or treated biofilms were analyzed with a one-way ANOVA followed by Tukey’s multiple comparison test.*

The effectiveness of aBL inactivation of the biofilms varied depending on the species. In monomicrobial biofilms, *P. aeruginosa* was the most susceptible to aBL with 6.30 log_10_ CFU reduction in 24-hour old biofilms after 500 J/cm^2^ aBL was delivered (*P* < 0.0001) (Table 1). Under the same aBL exposure, 3.48- and 2.33-log_10_ CFU reductions were observed in 24-hour old MRSA (*P* < 0.0001) and *C. albicans* biofilms (*P* = 0.0025), respectively.

In the 24-hour old dual-species biofilms with *P. aeruginosa* and *C. albicans*, 6.34-log_10_ CFU reduction was observed in *P. aeruginosa* when an exposure of 500 J/cm^2^ had been delivered. (*P* < 0.0001; Table 1), which was almost the same extent of inactivation as in the monomicrobial biofilms. However, *C. albicans* became more susceptible to aBL than in the monomicrobial biofilm and 3.11 log_10_ CFU reduction was observed (Table 1; *P* < 0.0001).

When the dual-species biofilms with *P. aeruginosa* and MRSA were exposed to 500 J/cm^2^ aBL, 2.37 log_10_ CFU reduction was observed in MRSA (*P* = 0.0019) and 3.40 log_10_ CFU reduction in *P. aeruginosa* (*P* < 0.0001) (Table 1).

#### 48-Hour Old Biofilms

After 48 h of incubation, the cell density in monomicrobial biofilms increased in all species in comparison to that of 24 h old biofilms (Table 2). In the dual-species biofilms with *P. aeruginosa* and *C. albicans*, the cell density of *P. aeruginosa* increased while the cell density of *C. albicans* decreased when the incubation time was increased from 24 to 48 h (Table 2). In the dual-species biofilms with *P. aeruginosa* and MRSA, the cell density of both species increased slightly (Table 2).

**Table 2:** Effectiveness of antimicrobial blue light (405 nm) inactivation of biofilms grown in 96-well microtiter plates after 48 h of incubation.

Biofilm type	Strain		Irradiance (mW/cm^2^)	Irradiation Time (min)	Radiant exposure (J/cm^2^)	Biofilm cell density Log_10_ (CFU/mL)	SE	Survival bacterial counts Log_10_(CFU/mL)		SE
Monomicrobial	*C. albicans*	CEC 749	60.0	60	216	8.41	0.06	-0.25	ns	0.26
			92.6	90	500	8.51	0.09	-2.11	^∗∗∗^	0.09
	MRSA	USA300	60.0	60	216	8.77	0.13	-1.62	^∗^	0.13
			92.6	90	500	9.97	0.37	-2.35	^∗∗∗^	0.23
	*P. aeruginosa*	IQ0046	60.0	60	216	9.34	0.23	-3.67	^∗∗∗^	0.73
			92.6	90	500	8.88	0.16	-6.88	^∗∗∗^	0.16
Polymicrobial	*C. albicans*	CEC 749	60.0	60	216	6.84	0.30	-2.96	^∗∗∗^	0.40
			92.6	90	500	6.02	0.28	-3.41	^∗∗∗^	0.21
	*P. aeruginosa*	IQ0046	60.0	60	216	9.45	0.18	-5.48	^∗∗∗^	0.17
			92.6	90	500	9.41	0.29	-7.41	^∗∗∗^	0.29
Polymicrobial	MRSA	USA 300	60.0	60	216	8.50	0.24	-2.44	^∗∗∗^	0.39
			92.6	90	500	8.44	0.09	-2.61	^∗∗∗^	0.08
	*P. aeruginosa*	IQ0046	60.0	60	216	8.80	0.12	-4.19	^∗∗∗^	0.10
			92.6	90	500	8.15	0.08	-3.67	^∗∗∗^	0.24

*ns, not significant; ^∗^P < 0.05, ^∗∗∗^ P < 0.001. SE, standard error. The differences between untreated or treated biofilms were analyzed with a one-way ANOVA followed by Tukey’s multiple comparison test.*

After treatment with 500 J/cm^2^ aBL, the CFU reductions in the monomicrobial biofilms of each species were 2.11-, 2.35-, and 6.88-log_10_ for *C. albicans* (*P* = 0.0009), MRSA (*P* < 0.0001), and *P. aeruginosa* (*P* < 0.0001), respectively. For the dual-species biofilms with *C. albicans* and *P. aeruginosa*, 3.41- and 7.41-log_10_ CFU reduction were observed after 500 J/cm^2^ aBL exposure (*P* < 0.0001) (Table 2), indicating that 1.30- and 0.53-log_10_ CFU more inactivation of *C. albicans* and *P. aeruginosa* were achieved in the dual-species biofilms than in the monomicrobial ones.

On the other hand, 2.61- and 3.67-log_10_ CFU reduction was obtained in MRSA (*P* < 0.0001) and *P. aeruginosa* (*P* < 0.0001), respectively, in the dual-species biofilms with the two species. In comparison to the monomicrobial biofilms of *P. aeruginosa*, aBL-induced reduction of *P. aeruginosa* decreased by 3.21-log_10_ CFU in the dual-species biofilms with MRSA and *P. aeruginosa*. In the case of MRSA, 0.26-log_10_ CFU more inactivation was observed in the polymicrobial biofilm with *P. aeruginosa* compared to the monomicrobial biofilm (Table 2).

The temperature was measured in the 96-well microtiter plates during 405-aBL exposure (216 and 500 J/cm^2^). The highest temperature recorded was 37.5°C, at which no loss of the viability of any of the microorganisms was observed.

### aBL Inactivation of Biofilms Grown in the CDC Biofilm Reactor

When the monomicrobial biofilms of each species were established in the CDC biofilm reactor under a turbulent flow of nutrients for 48 h, the cell densities of the biofilms were 7.36 log_10_ CFU/cm^2^ for *C. albicans*, 9.23 log_10_ CFU/cm^2^ for *P. aeruginosa* and 6.83 log_10_ CFU/cm^2^ for MRSA (Table 3). When the dual-species biofilms with *C. albicans* and *P. aeruginosa* were grown in the CDC biofilm reactor for the same time period, the cell densities changed to 5.83 log_10_ CFU/cm^2^ for *C. albicans* and 8.07 log_10_ CFU/cm^2^ for *P. aeruginosa* (Table 3). For the 48-h old dual-species biofilms with MRSA and *P. aeruginosa*, the cell densities were 7.44- and 7.52-log_10_ CFU/cm^2^ for MRSA and *P. aeruginosa*, respectively (Table 3).

**Table 3:** Antimicrobial blue light (405 nm) inactivation of biofilms grown in the CDC biofilm reactor after 48 h of incubation.

Biofilm type	Strain		Irradiance (mW/cm^2^)	Irradiation Time (min)	Radiant exposure (J/cm^2^)	Biofilm cell density Log_10_ (CFU/cm^2^)	SE	Survival bacterial counts Log_10_(CFU/cm^2^)		SE
Monomicrobial	*C. albicans*	CEC 749	60.0	60	216	7.25	0.08	-0.14	ns	0.26
			92.6	90	500	7.48	0.12	-1.04	ns	0.25
	MRSA	USA300	60.0	60	216	6.83	0.10	-2.05	ns	0.33
			92.6	90	500	6.83	0.10	-2.58	ns	0.30
	*P. aeruginosa*	IQ0046	60.0	60	216	9.52	0.92	-1.48	ns	0.14
			92.6	90	500	8.94	0.38	-3.70	^∗∗^	0.95
Polymicrobial	*C. albicans*	CEC 749	92.6	90	500	5.83	0.15	-1.71	ns	0.16
	*P. aeruginosa*	IQ0046	92.6	90	500	8.07	0.12	-2.58	ns	0.45
Polymicrobial	MRSA	USA300	60.0	60	216	7.44	0.20	-0.80	ns	0.57
			92.6	90	500	7.44	0.20	-0.86	ns	0.41
	*P. aeruginosa*	IQ0046	60.0	60	216	7.52	0.23	-1.80	ns	0.28
			92.6	90	500	7.52	0.23	-3.56	^∗∗^	1.07

*ns, no significant; ^∗∗^ P < 0.01. SE, standard error. The differences between untreated or treated biofilms were analyzed with a one-way ANOVA followed by Tukey’s multiple comparison test.*

When an aBL exposure of 216 J/cm^2^ had been delivered, 1.48- and 2.05-log_10_ CFU reduction were observed in *P. aeruginosa* and MRSA monomicrobial biofilms, respectively (ns, *P* > 0.05), while only minimal reduction of CFU was observed in *C. albicans* monomicrobial biofilms. When the aBL exposure was increased to 500 J/cm^2^, the aBL-induced inactivation of microorganisms in the respective monomicrobial biofilms were 1.04- (ns, *P* > 0.05), 2.58- (ns, *P* > 0.05) and 3.70-log_10_ CFU (*P* = 0.0017) for *C. albicans*, MRSA, and *P. aeruginosa*, respectively.

When the two species of *P. aeruginosa* and *C. albicans* were incubated together and formed dual-species biofilms, 2.58-log_10_ CFU inactivation of *P. aeruginosa* and 1.71-log_10_ CFU inactivation of *C. albicans* were observed after treatment with 500 J/cm^2^ aBL (ns, *P* > 0.05). In the dual-species biofilms with MRSA and *P. aeruginosa*, aBL-induced reduction of CFU was 3.56- log_10_ and 0.86-log_10_ for *P. aeruginosa* (*P* = 0.0029) and MRSA (ns, *P* > 0.05), respectively.

**FIGURE 1:**
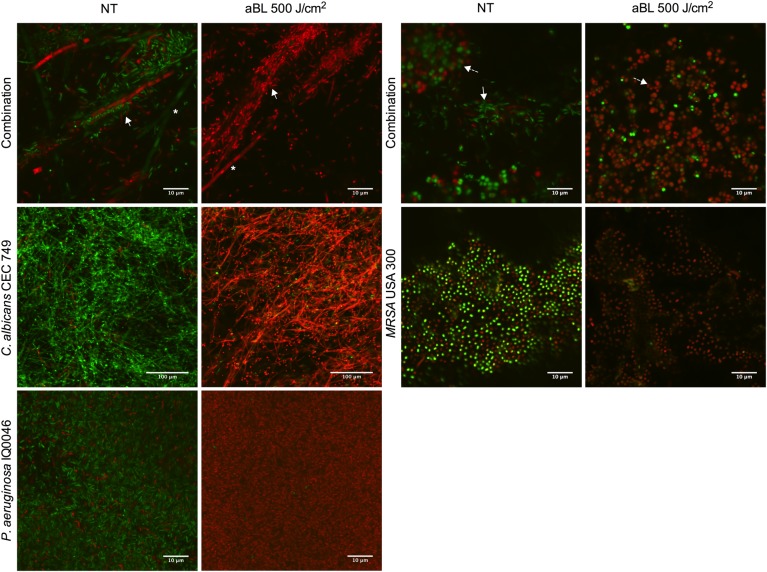
Confocal laser microscope images showing the cytotoxic activity of aBL in *P. aeruginosa* IQ0046, MRSA USA300 and *C. albicans* CEC 749 mono and polymicrobial biofilms. Scale bars represent 10 or 100 μm. NT, no treatment; aBL, biofilm treated with 405-nm aBL (92.6 mW/cm2, 500 J/cm^2^). In the polymicrobial biofilms, ^∗^ indicate *C. albicans* CEC 749, solid white arrows indicate *P. aeruginosa* IQ0046 and dashed line arrows indicate MRSA USA300.

Confocal laser scanning microscopy images confirmed that both monomicrobial and polymicrobial biofilms were successfully established in the CDC reactor (Figure 1). In all the untreated monomicrobial biofilms, spontaneously dead microorganisms (as stained red with PI) were observed. This event became more prevalent in the dual-species biofilms with *C. albicans* and *P. aeruginosa*, where some *C. albicans* hyphae also dyed with PI in the untreated biofilms. After an exposure of 500 J/cm^2^ aBL, almost all the microbial cells in the biofilms appeared dead, as evidence by the red staining with PI in the CLSM images (Figure 1).

**FIGURE 2:**
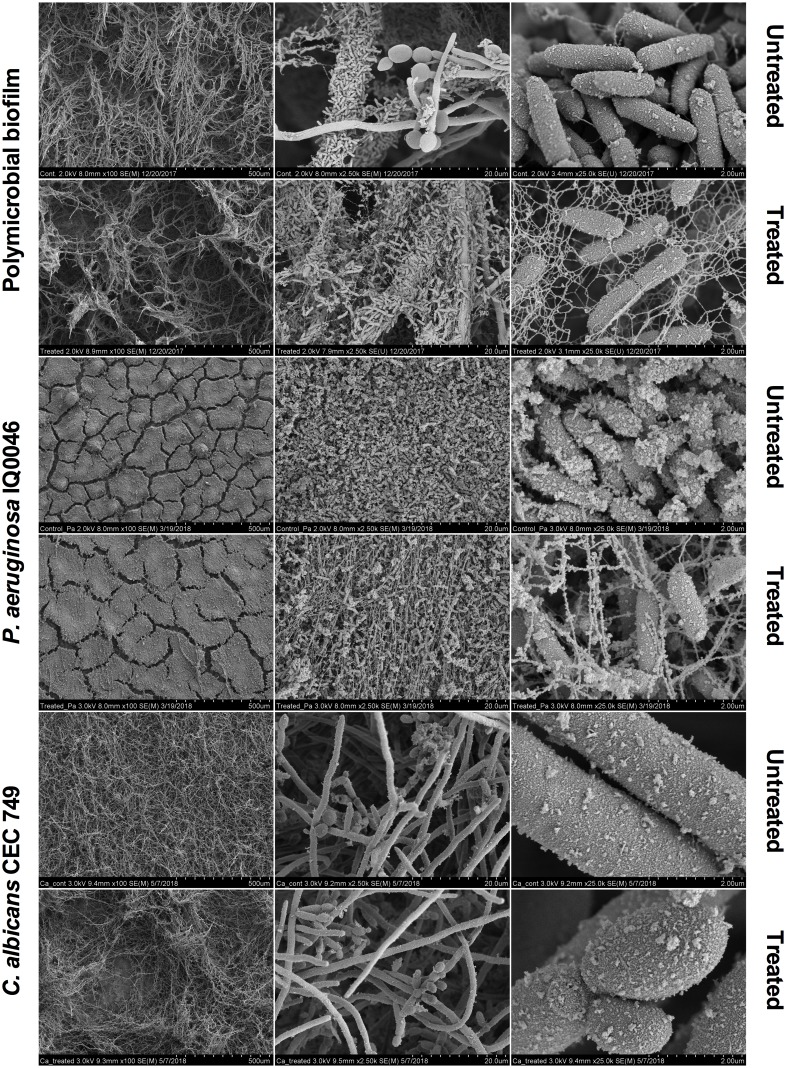
Scanning electron microscope images showing the cytotoxic activity of aBL at 405 nm in *P. aeruginosa* IQ0046 and *C. albicans* CEC 749 monomicrobial and polymicrobial biofilms after an aBL exposure of 500 J/cm^2^ (92.6 mW/cm^2^ and 90 min). Scale bars represent 500, 20, or 2 μm.

**FIGURE 3:**
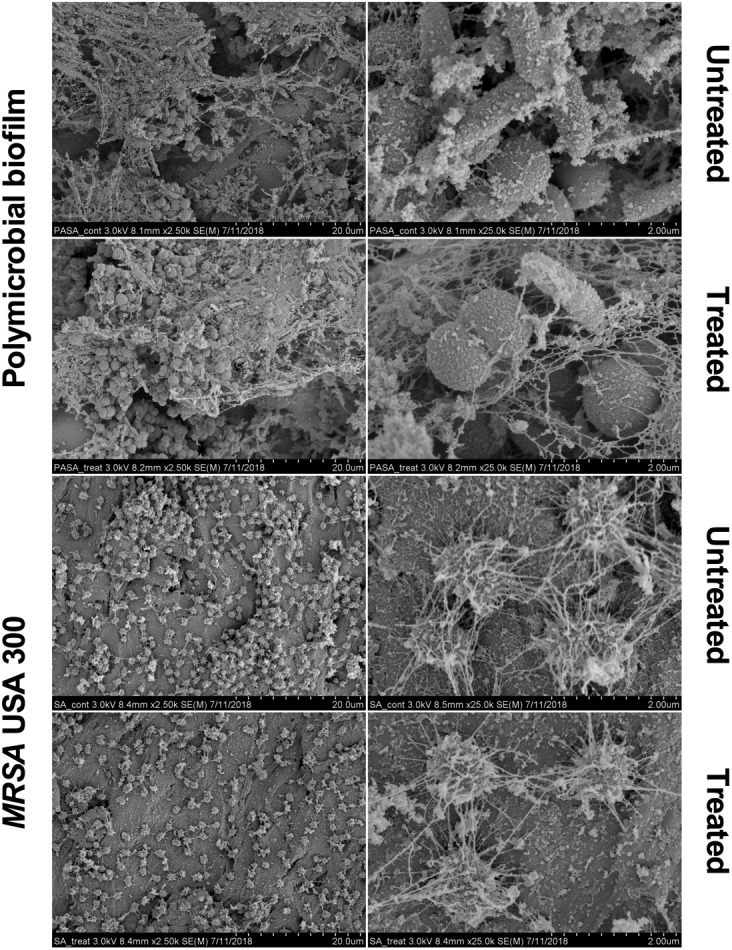
Scanning electron microscope images showing the bactericidal activity of aBL at 405 nm in *P. aeruginosa* IQ0046 and MRSA USA300 monomicrobial and dual-species microbial biofilms after an aBL exposure of 500 J/cm^2^ (92.6 mW/cm^2^ and 90 min). Scale bars represent 20 or 2 μm.

Scanning electron microscopy images revealed that aBL induced morphological changes in both the monomicrobial and polymicrobial biofilms (Figure 2, 3). Untreated *C. albicans* and *P. aeruginosa* monomicrobial biofilms showed highly dense and compact microbial cells (Figure 2). Untreated MRSA monomicrobial biofilm, on the other hand, had a smaller biofilm density and the surface of the polycarbonate coupon could be easily observed (Figure 3). All monomicrobial biofilms were surrounded with extracellular polymeric substance (EPS). *P. aeruginosa* and MRSA seemed to have a larger amount of EPS than *C. albicans*. The EPS of *P. aeruginosa* was organized like a mesh while the EPS of MRSA appeared in a star-like fashion. *C. albicans* biofilm was composed of yeast and hyphal forms.

After an aBL exposure of 500 J/cm^2^ the density of the microbial cells in the monomicrobial biofilms decreased in all three species. The cell density decrease in *C. albicans* and MRSA monomicrobial biofilms was better observed with × 100 or × 2.5 k magnification. For *P. aeruginosa* monomicrobial biofilm, on the other hand, the decrease of cell density after aBL treatment was much more evident under × 25.0 k magnification; although, they can also be observed in all the other magnifications.

In agreement with the CLSM images, *P. aeruginosa* attached to the surface of *C. albicans* in the dual-species biofilm and formed a very dense biofilm with lower amount of EPS than observed in the monomicrobial biofilms. The effect of the aBL treatment on the *P. aeruginosa* and *C. albicans* dual-species biofilm could be observed in the decrease in microbial cell density under × 100 magnification for *C. albicans* and under × 25.0 k magnification for *P. aeruginosa* (Figure 2).

The SEM images of the dual-species biofilm with MRSA and *P. aeruginosa* revealed that the two microorganisms are infiltrated together (Figure 3). Similar to the dual-species biofilm with *C. albicans* and *P. aeruginosa*, the cell density of the dual-species biofilm with MRSA and *P. aeruginosa* decreased after treatment with 500 J/cm^2^ aBL (92.6 MW/cm^2^ and 90 min) (× 2.5 k magnification). In addition, *P. aeruginosa* and MRSA dual-species biofilm had lower amount and thinner EPS than the monomicrobial biofilms (Figure 3).

The temperature in the 35-mm petri dishes was measured during 405-aBL exposure (216 J/cm^2^ and 500 J/cm^2^). The highest temperature recorded was 33.1°C.

### Phototoxicity of aBL to Normal Human Keratinocytes

**FIGURE 4:**
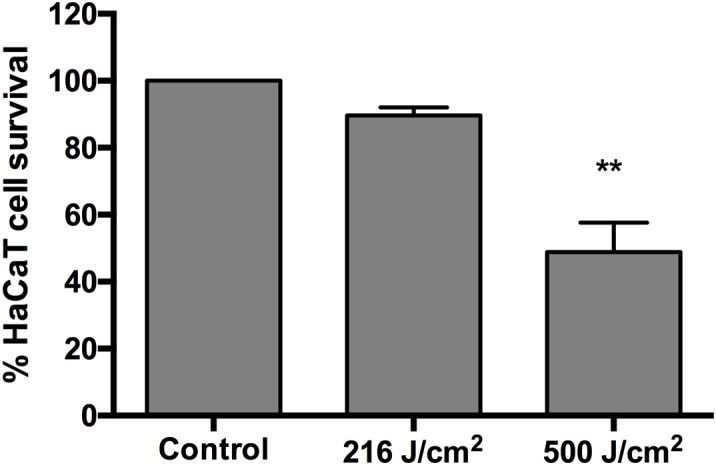
Cytotoxicity of 405-nm aBL to normal human HaCaT cells after an exposure of 216 J/cm^2^ (60 mW/cm^2^, 60 min) or 500 J/cm^2^ (92.6 mW/cm^2^, 90 min). 0 vs 216 J/cm^2^ (*P* = 0.4016) and 0 vs 500 J/cm^2^ (*P* = 0.0011). Bars are the SE. ^∗∗^*P* < 0.01.

The phototoxicity study of aBL showed no statistically significant loss of viability in HaCaT cells after an exposure of up to 216 J/cm^2^ (60 mW/cm^2^, 60 min) (*P* = 0.4016) (Figure 4). The exposure of HaCaT cells to 500 J/cm^2^ (92.6 mW/cm^2^, 90 min) reduced the metabolism of the cells significantly (*P* = 0.0011) to 49% of the unirradiated control (Figure 4).

## Discussion

In this study, we investigated the efficacy of 405 nm aBL for the treatment of *P. aeruginosa*, MRSA, and *C. albicans* in monomicrobial and polymicrobial biofilms formed in 96-well microtiter plates and the CDC biofilm reactor. Our findings demonstrated that in 96-well microtiter plates both the monomicrobial and polymicrobial biofilms (either 24 or 48 h old) were highly susceptible to 405 nm aBL. When the biofilms were developed in the CDC biofilm reactor, they exhibited higher tolerance to aBL. A possible reason for that result is the increased thickness of the biofilms obtained in the CDC biofilm reactor. Both techniques used in this study have their advantages and limitations. Microtiter plates are high-throughput and inexpensive but they suffer an exhaustion of nutrients and loosely attached biofilm can be detached during washing steps. The CDC biofilm reactor, on the other hand, is low-throughput and requires special equipment, but in this case, biofilms are grown with continuous supply of fresh medium and under shear stress (Azeredo et al., 2017), which is more clinically relevant. Hydrodynamics have major impact on biofilm growth, and biofilms grown under higher concentrations of nutrients and higher shear stress will be more abundant, filamentous and have higher cell density and thicker and more complex EPS. Those grown under lower nutrient concentrations and lower shear stress will be more compact with a lower cell density of a single layer of microcolonies and have thinner EPS. In addition, an open biofilm structure in the CDC biofilm reactor will facilitate the diffusion of nutrients easier than in the compact structure (Molobela and Ilunga, 2011).

Under most of the conditions, the CFU reduction by aBL in the *C. albicans* and *P. aeruginosa* dual-species biofilms was higher than that in the respective monomicrobial biofilms. Conversely, the CFU reduction by aBL obtained in the dual-species biofilms with MRSA and *P. aeruginosa* was less than that obtained in the respective monomicrobial biofilms. This seemed to be in relation with the different kind of interactions between these specific pathogens. The results from the present study showed that an antagonistic interaction between *C. albicans* and *P. aeruginosa* in the dual-species biofilms favored aBL inactivation and a synergistic interaction between MRSA and *P. aeruginosa* appeared to hinder aBL inactivation.

To the best of our knowledge, this study is the first to investigate the activity of aBL at 405 nm against biofilms using the *in vitro* dynamic biofilm model CDC reactor and the effect of aBL at 405 nm against dual-species biofilms with *P. aeruginosa* and MRSA as well as with *P. aeruginosa* and *C. albicans*. Other polymicrobial biofilms where aBL has been tested include dual-species biofilms formed by *E. coli* and *S. aureus*, in which 1.2 and 1.7 log_10_ CFU/mL inactivation were obtained, respectively, after 254.66 J/cm^2^ aBL at 405 nm was delivered (McKenzie et al., 2013) and *in vivo* human periodontal polymicrobial biofilms, in which an inactivation of 0.25 and 0.56 log_10_ CFU/mL in *Porphyromonas gingivalis* and *Prevotella intermedia* was obtained, respectively, after 8.45 J/cm^2^ aBL at 455 nm was delivered daily for 4 days (Soukos et al., 2015). In addition, a previous study showed that, after an exposure of 540 J/cm^2^ at 415 nm, no apoptotic cells developed in aBL-irradiated mouse skin (Wang et al., 2016).

The results presented in the CLSM (Figure 1) and SEM (Figure 2, 3) images as well as the cell densities of the monomicrobial and polymicrobial biofilms observed in the microtiter plates (Tables 1, 2) and in the CDC biofilm reactor (Table 3) demonstrate the interactions between different microbial species in the polymicrobial biofilms. As described previously, *P. aeruginosa* formed its biofilm in the hyphae of *C. albicans* and as a result of that association, the hyphae of the fungus are damaged (Peleg et al., 2010). This resulted in reduced number of *C. albicans* CFU after 48 h of incubation with *P. aeruginosa* (Tables 2, 3). In addition, the susceptibility of *C. albicans* to aBL increased in dual-species biofilms probably due to a synergistic effect with the damage to *C. albicans* cells induced by *P. aeruginosa*. On the other hand, the SEM images of the dual-species biofilms with MRSA and *P. aeruginosa* revealed that the two microorganisms are infiltrated together (Figure 3). The interaction between the two species seems to be synergistic as *P. aeruginosa* promotes MRSA adherence to the substrate in the turbulent environment of the CDC reactor. This is evidenced by the increase in MRSA cell density in the dual-species biofilms in comparison to the monomicrobial biofilm, and the decrease in EPS production observed in the SEM images (Table 3 and Figure 3). In addition, the tolerance of *P. aeruginosa* to aBL treatment increased when cocultured with MRSA. This can be attributed to the production of carotenoid pigments by MRSA, which are known to physically quench singlet oxygen and to protect bacteria from the lethal effects of aBL (Dahl et al., 1989; Glaeser et al., 2011).

As was reported in previous studies (Murdoch et al., 2013), aBL inactivation of microorganisms is oxygen dependent. Oxygen availability can be a concern in biofilms as actively respiring aerobic microcolonies can consume oxygen faster than oxygen diffusion, which results in the formation of anaerobic zones in the deep layers in the deep layers of the biofilm (Flemming et al., 2016). This explains the finding in the present study that the biofilms grown in the CDC biofilm reactor are more tolerant to aBL inactivation than the biofilms grown in the 96-well microplates. As the biofilms obtained in the CDC biofilm reactor are thicker than the biofilms grown in the 96-well microplates, oxygen availability has a bigger impact on them. A potential way to increase the effectiveness of aBL inactivation of biofilms is to combine aBL with hyperbaric oxygen therapy. It was reported that hyperbaric oxygen therapy, in which the air pressure is several times higher than normal air pressure, could increase oxygen diffusion depth in biofilms and, subsequently, facilitate ciprofloxacin killing (Gade et al., 2018).

As shown in Tables 1, 2, an aBL exposure of 216 J/cm^2^ reduced the viability of the polymicrobial biofilms very significantly. In contrast, the phototoxicity study of aBL showed no statistically significant loss of viability in HaCaT cells after an exposure of up to 216 J/cm^2^ (Figure 4). Significant cytotoxicity was observed in the HaCaT cells after an exposure of 500 J/cm^2^ aBL.

The antimicrobial effect of aBL could be further improved synergizing its activity with a photosensitizer or antibiotic treatment. A synergistic combination could allow a decrease in the exposure time and radiant exposure, reducing the phototoxicity to the host cells and widening the therapeutic window of aBL.

The results obtained in the present study indicate the promising efficacy of aBL inactivation of polymicrobial biofilms, for treatment of infections in which multiple antimicrobials are usually required in order to eradicate different microbial species. Future studies are warranted to investigate other important biofilm-forming microbial species, the strategies to enhance the effectiveness of aBL, and aBL treatment of polymicrobial biofilms-related infections *in vivo*.

## Author Contributions

RF-E, and TD designed the study. RF-E, XL, and XG performed the experiments. RF-E and TD analyzed the data and wrote the manuscript.

## Conflict of Interest Statement

The authors declare that the research was conducted in the absence of any commercial or financial relationships that could be construed as a potential conflict of interest.
